# Severe and Fatal Multilobar Nonclassic Radiation Pneumonitis following Stereotactic Body Radiation Therapy (SBRT) for Treatment of Inoperable Non-Small-Cell Lung Cancer: A Report of Two Cases and Possible Enhancement by Concurrent Amiodarone

**DOI:** 10.1155/2019/8754951

**Published:** 2019-08-04

**Authors:** Anastasios Georgiou, Adam Farmer

**Affiliations:** ^1^Radiation Oncology, Carl Kirkland Cancer Center, 620 W. Forest Ave, Jackson, TN 38301, USA; ^2^Carl Kirkland Cancer Center, 620 W. Forest Ave., Jackson, TN 38301, USA

## Abstract

Stereotactic body radiation therapy (SBRT) is considered the standard of care for treatment of inoperable early stage non-small cell carcinoma of the lung. SBRT delivers a very high dose of ionizing radiation to a relatively small region encompassing the tumor and spares a significant portion of the remaining lung from high doses. However, the conformal high dose comes at the expense of treating a larger volume of normal lung to lower doses. In general, this has been deemed to be acceptable with an overall lower risk of radiation pneumonitis. However, in the face of predisposing factors, the higher doses delivered by this technique may lead to an increase in radiation pneumonitis. We report on two patients being treated with SBRT in which severe radiation pneumonitis developed in spite of our radiation dosimetry being significantly below the acceptable limit for lung toxicity. Both patients developed a “fulminant” form of radiation pneumonitis with radiographic abnormalities well beyond the treated volume. In one patient, the disease proved fatal. Both patients were on amiodarone at the time SBRT was administered. Given the rarity of fulminant radiation pneumonitis, especially with the relatively small fields treated by SBRT, we suspect that amiodarone enhanced the pulmonary toxicity.

## 1. Introduction

Stereotactic body radiation therapy (SBRT) is now considered the standard of care for treatment of inoperable early stage non-small cell carcinoma of the lung [1]. SBRT delivers a very high dose of ionizing radiation to a relatively small region encompassing the tumor and spares a significant portion of the remaining lung from high doses. This can be achieved by a variety of techniques with Volumetric Modulated Arc Therapy (VMAT), an advanced form of Intensity Modulate Radiation Therapy (IMRT) that dynamically delivers a precisely-sculpted 3D dose distribution with a 360-degree rotation of the linear-accelerator gantry in a single or multi-arc treatment, being an accepted form of delivery of the dose. However, the conformal high dose comes at the expense of treating a larger volume of normal lung to lower doses [2]. In the absence of predisposing factors, the use of VMAT may result in a decrease in the risk of radiation pneumonitis [3, 4]. However, even with the use of highly conformal techniques, patients with predisposing factors such as contralateral pneumonectomy [5], immunosuppression[6], administration of concurrent chemotherapy [7], and interstitial lung disease[8, 9] may be at increased risk for radiation pneumonitis.

We present two patients treated with VMAT based SBRT who developed severe bilateral pulmonary infiltrates highly suggestive of radiation pneumonitis. Of the two patients, one rapidly deteriorated and the process proved fatal. In both cases, the degree of radiographic abnormality was out of proportion to the expected pattern of radiation pneumonitis especially since both patients received SBRT. The doses to the normal surrounding lung were well within the accepted limits. However, given the striking radiographic abnormalities that were noted in all lung fields, even those receiving very little radiation exposure, a review was made to identify any previously unknown predisposing factors.

With the exception of both patients being on amiodarone, a known pulmonary toxin, other contributing etiologies were ruled out as best as possible. A review of the literature led to a finding that amiodarone is also a photosensitizer. Furthermore, there have been a few older case reports in which amiodarone was suspected of enhancing radiation mucositis. To our knowledge, there are no reports of amiodarone enhanced radiation pulmonary toxicity. However, this may be due to the fact that up until recently, the majority of patients have been treated with techniques giving much lower radiation doses to large volumes of the lung and with much lower doses per fraction than those administered via SBRT.

## 2. Case Presentation

The authors note that written informed consent was obtained agreeing to publication of the following two cases.

### 2.1. Patient 1

An 81-year-old female presented with increasing cough and a chest x-ray revealed a right lower lung mass. Subsequent work-up with CT scans, PET scan, and needle core-biopsy yielded a diagnosis of a clinical stage T1cN0M0 adenocarcinoma of the lung. Her past medical history was pertinent in that she had chronic atrial fibrillation for which she had undergone cardiac ablation and placement of a permanent pacemaker. Pulmonary function studies revealed an FEV1 of 0.97 liters which was 56% of predicted. Her medications included allopurinol 300mg daily, atenolol 50mg daily, diltiazem extended release 180mg daily, furosemide 40mg daily, rivaroxaban 15mg daily and, amiodarone 200mg twice daily. The patient had been on amiodarone for at least three years and after first-line therapy for atrial fibrillation had been ineffective.

**Figure 1:**
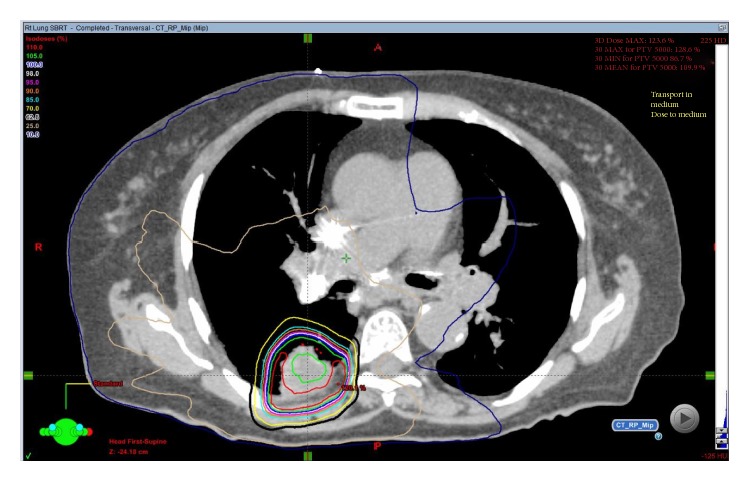
SBRT plan for Patient 1 showing conformal radiation dose delivery to the right pulmonary mass.

**Figure 2:**
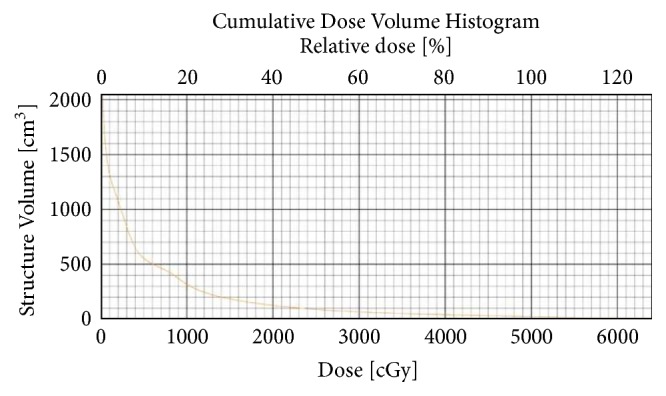
DVH showing total lung minus target dose for patient 1.

After presentation at a multi-disciplinary lung cancer conference, it was decided that she was not a surgical candidate. Therefore, she was treated with a total of 50Gy in 5 fractions of SBRT using two co-planar arcs (Figure 1). The Dose-Volume Histogram (DVH) for the treatment plan showed total lung minus target dose within accepted guidelines (Figure 2).

**Figure 3:**
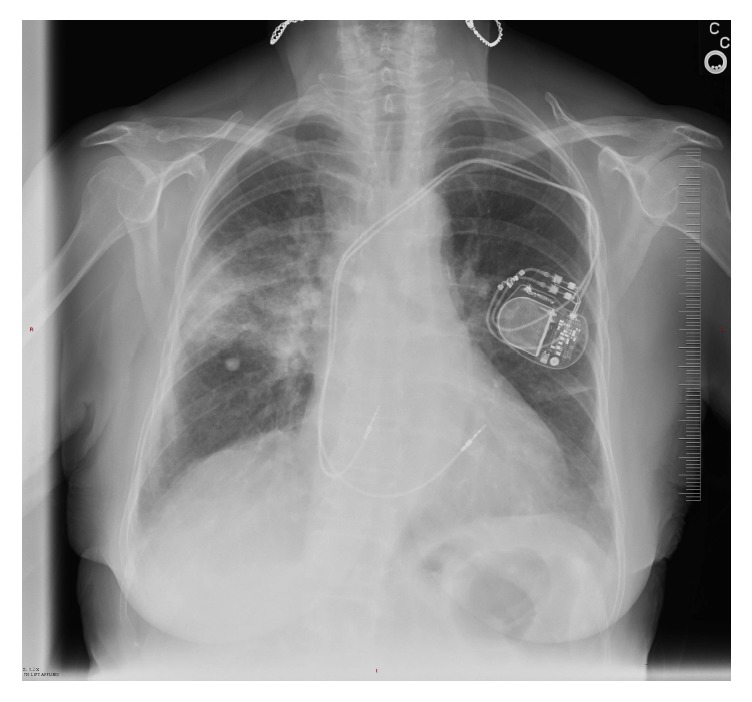
A chest X-ray showing the development of lung infiltrates in and around the treated right lung mass occurring one month following completion of SBRT.

One month later, she presented with mild dyspnea and a nonproductive cough. She denied fevers, chills, hemoptysis, weight loss, or chest pain. Her physical examination did reveal coarse rales in the right lung field. A chest x-ray at that time revealed a new right upper lung infiltrate at the treated site (Figure 3). Her laboratory work was unremarkable with no elevation of the white blood cell count. A diagnosis of radiation pneumonitis was made and she was placed on prednisone 20 milligrams b.i.d.

**Figure 4:**
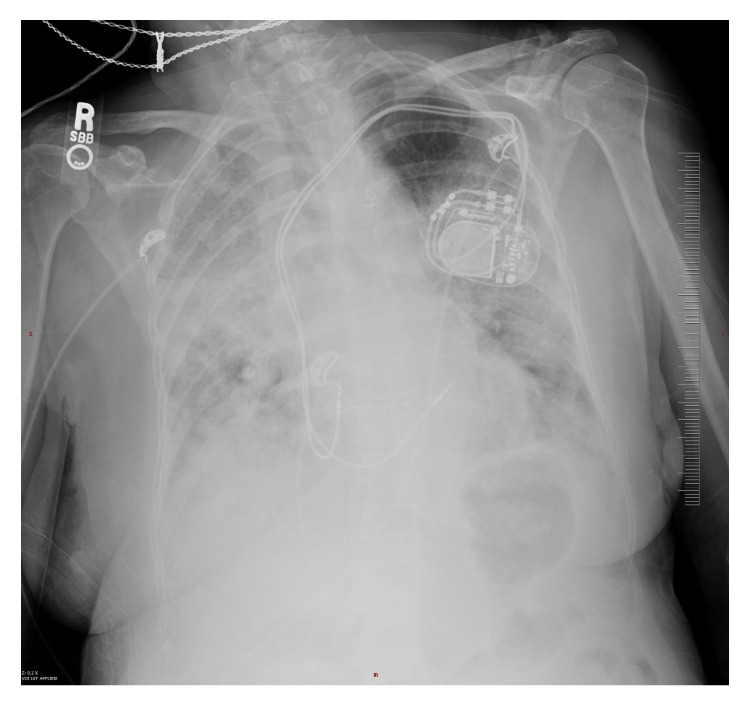
A chest X-ray, taken 5 days after the chest X-ray depicted in Figure 3, revealing rapid progression of lung infiltrates in multiple lobes.

Unfortunately, she presented 5 days later to the emergency room with increasing dyspnea and nonproductive cough. A chest x-ray now revealed bilateral pulmonary infiltrates (Figure 4). There had been marked worsening of the infiltrates in the right lung. The patient's respiratory status declined and she required intubation and mechanical ventilation

**Figure 5:**
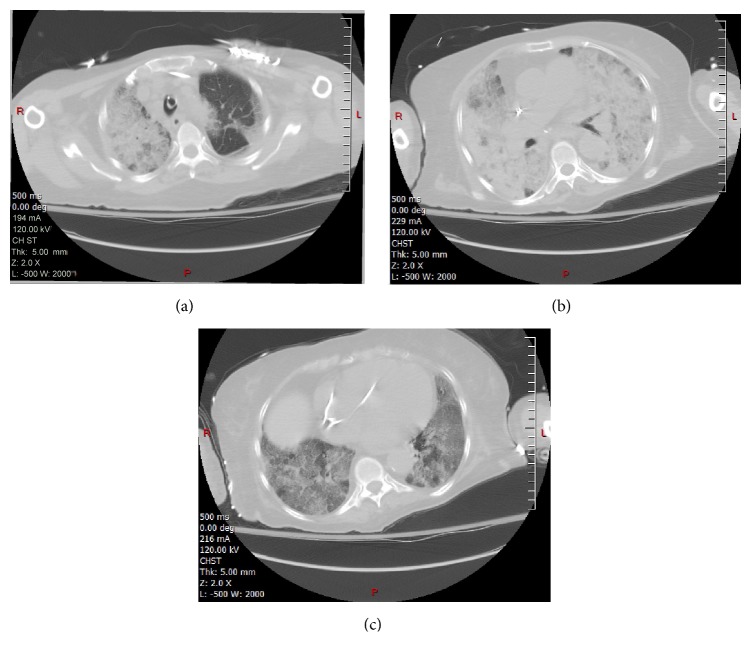
CT scan images above the plane, thru the plane, and below the plane of SBRT treatment showing diffuse infiltrates ((a), (b), and (c) images respectively).

A nuclear medicine ventilation/perfusion scan revealed a low probability for pulmonary emboli. A CT scan of the chest revealed diffuse bilateral pulmonary infiltrates in all lobes of both lungs (Figure 5).

Urine tests for Streptococcus Pneumonia antigen, Histoplasma antibody, Blastomycosis antibody, and Legionella antigen were negative. Serum tests for Cryptococcal antigen, Histoplasma antibody, Coccidioides antibody, Blastomycosis antibody, and Aspergillus antibody were negative. Blood cultures and urine cultures were negative. A respiratory viral panel was negative.

She was treated with intravenous methylprednisolone. Empiric intravenous antibiotic and antifungal therapy was started with doxycycline, meropenem, vancomycin, and caspofungin. Her rivaroxaban was discontinued in preparation for fiber-optic bronchoscopy once the risk of bleeding during the procedure had subsided. Her amiodarone was not discontinued.

In spite of maximal support, she succumbed to her disease within 72 hours after her initial presentation with the suspected radiation induced changes and prior to evaluation with bronchoscopy.

### 2.2. Patient 2

A 69-year-old gentleman initially presented with left-sided chest pain and upper abdominal pain. A CT scan of the chest was performed that revealed a 4.3 cm mass in the posterior left lower lung. A needle core biopsy revealed a moderately differentiated squamous cell carcinoma. Staging studies included a PET and MRI of the brain. This gentleman's disease was staged as a clinical T2N0M0 lung cancer.

This gentleman's past medical history was significant for a prior stroke in the left middle cerebral artery region leaving him with right-sided weakness. He had significant coronary artery disease and had undergone prior coronary artery bypass grafting. His medications included aspirin 81mg daily, tamsulosin 0.4mg, levothyroxine 100mcg daily, lisinopril 20mg daily, lovastatin 40mg daily, clopidogrel 75mg daily, hydrochorothiazide 25mg- triamterene 37.5mg daily, and amiodarone 200mg daily. Amiodarone had been started two years prior due to ventricular arrhythmia.

**Figure 6:**
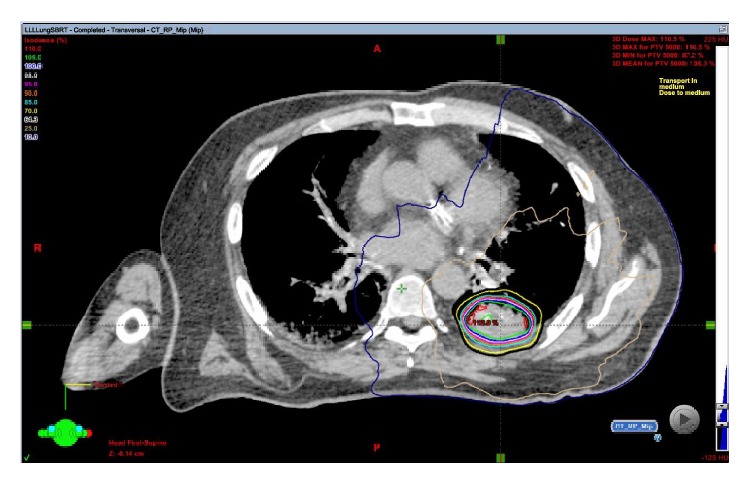
SBRT plan for Patient 2 showing conformal radiation dose delivery to the left pulmonary mass.

**Figure 7:**
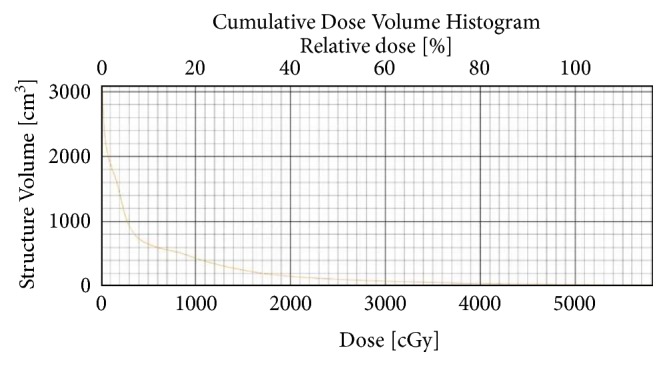
DVH showing total lung minus target dose for patient 2.

After discussion with his cardiologist and pulmonologist, this gentleman was referred for radiation therapy. Consequently, he received a total dose of 50Gy in 5 fractions using SBRT thru two coplanar arcs (Figure 6). The Dose-Volume Histogram (DVH) for the treatment plan showed total lung minus target dose within accepted guidelines (Figure 7).

A CT scan of the chest performed 3 months after completion of therapy revealed a significant decrease in the size of the left lower lung mass with no evidence to suggest progression of disease. A PET scan performed 6 months later revealed complete resolution of the previously identified hypermetabolic mass in the left lower lung.

Nine months after his treatment, he presented with acute onset of confusion. He was noted to have significant hypoxemia on room air that required high-flow nasal cannula at 6 liters/minute. His arterial blood gases showed a pH of 7.45, pCO2 of 31, PO2 56. His white count was normal. A nuclear medicine ventilation perfusion scan was performed that revealed no evidence of pulmonary emboli.

**Figure 8:**
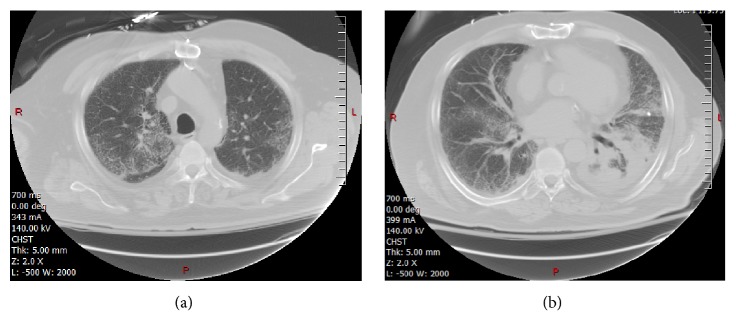
CT images above and at the plane of SBRT treatment showing lung infiltrates and dense consolidation in the left lower lung ((a) and (b) images respectively).

A CT scan of the chest was performed that revealed volume loss in both lungs with coarse subpleural interstitial changes in the upper and lower lobes (Figure 8). Bibasilar atelectasis and pneumonitis greater in the left lung base were noted.

Urine tests for Streptococcus Pneumoniae and Legionella were negative. A respiratory viral panel was negative. Blood and urine cultures were negative. Fiber-optic bronchoscopy was performed and biopsies from the left lower lung region revealed mild inflammation. The biopsied portions of pulmonary parenchyma showed interstitial fibroblastic proliferation with fibrinous material. The pathologist commented these findings might be observed in the case of organizing/fibrous pneumonia. The bronchial brushings from left lower lung showed significant atypia favoring squamous cell carcinoma. However the bronchial washings were negative for malignancy.

He was treated empirically with intravenous levofloxacin and steroids. His amiodarone was discontinued. His pulmonary status slowly improved and he was able to avoid intubation. Given his debilitated state, the patient chose surveillance.

**Figure 9:**
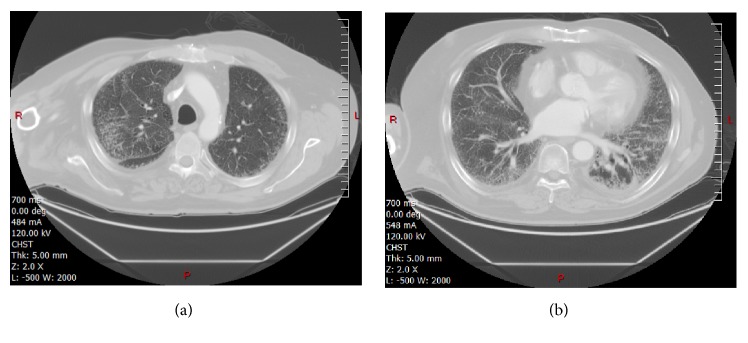
CT images 3 months after the initial presentation of pneumonitis. Images are at approximately the same level as those shown in Figure 6. There has been considerable clearing of the infiltrates and dense consolidation.

A CT scan of the chest performed 3 months after his hospitalization revealed no evidence to suggest progression of cancer (Figure 9). There was improvement in the previously noted interstitial and posttreatment changes throughout the lung fields.

This gentleman is alive 18 months later after his initial presentation with dyspnea and the images taken in Figure 6. He remains dyspneic but a follow CT of the chest shows continued improvement in the infiltrates and consolidation. In retrospect, in spite of the cytology report favoring malignancy, it is doubtful that this gentleman had recurrence of disease. The findings on the cytology most likely represent significant atypia secondary to treatment effect. Either untreated lymphangitic carcinomatosis or local recurrence leads to a very poor survival with no expectation that the radiographic abnormalities would improve.

## 3. Discussion

Given the rarity of bilateral fulminant radiation pneumonitis, a review was undertaken to evaluate for any common predisposing factors in the two cases. It was noted that both patients were on amiodarone which is a known pulmonary toxin. A possible interaction between VMAT based SBRT lung cancer treatment and amiodarone warrants further scrutiny in light of the following issues.

### 3.1. Fulminant Sporadic Radiation Pneumonitis

Radiation pneumonitis is a known complication after radiation therapy for treatment of lung malignancy. The classical presentation usually occurs within 6 months of treatment with most cases occurring within 1 to 3 months after completion of thoracic radiotherapy. It is usually associated with symptoms of a nonproductive cough and dyspnea. Radiographs of the chest usually reveal pulmonary infiltrates within the irradiated portion of the lungs [10].

However, a more fulminant form of radiation pneumonitis has been reported in the literature. This has been referred to as “sporadic” radiation pneumonitis [6, 11]. This form is considered rare and therefore descriptions are confined to case reports. Unlike the more classic pattern, there are significant pulmonary infiltrates found throughout portions of the lung fields receiving either no or very little exposure to radiation therapy. This form of radiation pneumonitis may be an immune related phenomenon similar to a hypersensitivity reaction.

Given its rarity, the authors doubt that both of our reported cases of possible fulminant radiation pneumonitis are a coincidence without any common factors.

### 3.2. Amiodarone Toxicity

Amiodarone is a known pulmonary toxin. The most significant factors for amiodarone pulmonary toxicity are age of the patient, duration of therapy, and dose [12]. The onset of the disease consists of progressive generalized weakness, cough, and dyspnea. In its most severe form, amiodarone pulmonary toxicity can present as an Acute Respiratory Distress Syndrome (ARDS) with rapid onset of progressive diffuse pneumonitis and respiratory failure [13, 14].

It is well documented that amiodarone can lead to cutaneous photosensitivity [15–19]. Although the wavelength of ultraviolet light thought to cause photosensitivity reactions [20] is usually considered to be nonionizing, it should be noted that there is no absolute threshold distinguishing nonionizing from ionizing radiation in this portion of the electromagnetic spectrum.

In addition to its well-known pulmonary toxicity and lesser known toxicity of photosensitivity, amiodarone has been suspected of enhancing the mucosal side effects of therapeutic radiation. Several case reports are noted in the literature in which amiodarone was suspected of leading to severe mucosal and cutaneous toxicity out of proportion to that expected from radiation therapy for treatment of head and neck cancer [21–24].

It is noted that the patient with fatal pneumonitis was on amiodarone 200mg twice daily whereas the surviving second patient was on a lower dose of 200mg daily.

### 3.3. Dose Constraints for VMAT Based Treatment of Lung Cancer

For lung cancer patients, the use of VMAT allows the treating oncologist to better meet the* traditional* (emphasis added) dose constraints on lung volume [25]. Recently, there has been concern that new dose constraints needed for VMAT based therapy given a possible increase in fatal radiation pneumonitis [26]. In this regard, radiation oncologists and medical physicists have recognized that SBRT treatment via VMAT does lead to an increase in the volume of lung receiving low doses of ionizing radiation. However, in the two cases presented above, the radiographic changes were multilobar and included portions of the lungs that were clearly outside the coplanar fields used. Therefore, VMAT cannot be considered as the sole contributor to the wider effect seen in these two patients. However, in the presence of amiodarone, it is plausible that larger lung volumes exposed to low dose ionizing radiation through the use of VMAT may trigger an immune mediated response.

A review was made of the dose distribution in both patients. The radiation exposure to the normal lung was well within the accepted traditional limits for radiation induced pneumonitis.

## 4. Conclusion

In summary, given that the use of VMAT (or by analogy IMRT type of external beam treatment) exposes a larger volume of the normal lung to low doses of radiation therapy, coupled with amiodarone being a known pulmonary toxin and possible radiation-sensitizer, a plausible argument can be made that this combination of factors may lead to severe pulmonary toxicity. Given the increasing use of SBRT, the possibility of amiodarone induced severe radiation pneumonitis in patients being treated with this modality needs to be further investigated and documented. By reporting these two cases, our hope is that awareness of this possible interaction by pulmonologists and radiation oncologists will lead to scrutiny of past and future cases of severe fulminant radiation pneumonitis to further delineate any association between SBRT and amiodarone.
